# Supplementary material for: Construction and integrated analysis of crosstalking ceRNAs networks in laryngeal squamous cell carcinoma
Source: PeerJ. 2019 Jul 22;7:e7380. doi: 10.7717/peerj.7380 (PMC6657684; doi:10.7717/peerj.7380)
Supplement: Table S1 — All RNA-seq data used in the study come from these cases. Case ID, primary site, and gender are in this table. [file peerj-07-7380-s002.docx]

Supplemental Table 1. Case ID of raw data used in the study

|  | Case ID | Project | Primary Site | Gender |
| --- | --- | --- | --- | --- |
| 1 | TCGA-DQ-5629 | TCGA-HNSC | Larynx | Male |
| 2 | TCGA-CV-7437 | TCGA-HNSC | Larynx | Male |
| 3 | TCGA-CN-6992 | TCGA-HNSC | Larynx | Male |
| 4 | TCGA-CN-6021 | TCGA-HNSC | Larynx | Female |
| 5 | TCGA-CN-6022 | TCGA-HNSC | Larynx | Male |
| 6 | TCGA-CV-7440 | TCGA-HNSC | Larynx | Male |
| 7 | TCGA-BA-6870 | TCGA-HNSC | Larynx | Female |
| 8 | TCGA-CV-7410 | TCGA-HNSC | Larynx | Male |
| 9 | TCGA-HD-7229 | TCGA-HNSC | Larynx | Male |
| 10 | TCGA-CN-6997 | TCGA-HNSC | Larynx | Male |
| 11 | TCGA-CN-4727 | TCGA-HNSC | Larynx | Male |
| 12 | TCGA-CN-6023 | TCGA-HNSC | Larynx | Male |
| 13 | TCGA-CN-A641 | TCGA-HNSC | Larynx | Male |
| 14 | TCGA-BA-4076 | TCGA-HNSC | Larynx | Male |
| 15 | TCGA-CN-4723 | TCGA-HNSC | Larynx | Male |
| 16 | TCGA-CV-7433 | TCGA-HNSC | Larynx | Male |
| 17 | TCGA-CV-7177 | TCGA-HNSC | Larynx | Female |
| 18 | TCGA-CN-5355 | TCGA-HNSC | Larynx | Male |
| 19 | TCGA-CN-5356 | TCGA-HNSC | Larynx | Male |
| 20 | TCGA-CV-7415 | TCGA-HNSC | Larynx | Male |
| 21 | TCGA-CN-4735 | TCGA-HNSC | Larynx | Male |
| 22 | TCGA-CN-6988 | TCGA-HNSC | Larynx | Male |
| 23 | TCGA-CN-5363 | TCGA-HNSC | Larynx | Male |
| 24 | TCGA-CR-7398 | TCGA-HNSC | Larynx | Female |
| 25 | TCGA-BA-6868 | TCGA-HNSC | Larynx | Male |
| 26 | TCGA-CV-5441 | TCGA-HNSC | Larynx | Male |
| 27 | TCGA-CN-6012 | TCGA-HNSC | Larynx | Male |
| 28 | TCGA-CV-5444 | TCGA-HNSC | Larynx | Male |
| 29 | TCGA-D6-A74Q | TCGA-HNSC | Larynx | Male |
| 30 | TCGA-CN-A6V3 | TCGA-HNSC | Larynx | Male |
| 31 | TCGA-UF-A71D | TCGA-HNSC | Larynx | Female |
| 32 | TCGA-H7-A6C5 | TCGA-HNSC | Larynx | Male |
| 33 | TCGA-CN-6989 | TCGA-HNSC | Larynx | Male |
| 34 | TCGA-CV-5443 | TCGA-HNSC | Larynx | Male |
| 35 | TCGA-D6-6826 | TCGA-HNSC | Larynx | Female |
| 36 | TCGA-CV-7422 | TCGA-HNSC | Larynx | Female |
| 37 | TCGA-UF-A718 | TCGA-HNSC | Larynx | Male |
| 38 | TCGA-CV-5440 | TCGA-HNSC | Larynx | Male |
| 39 | TCGA-D6-A6EK | TCGA-HNSC | Larynx | Male |
| 40 | TCGA-CR-7370 | TCGA-HNSC | Larynx | Female |
| 41 | TCGA-CV-7245 | TCGA-HNSC | Larynx | Male |
| 42 | TCGA-UF-A7JF | TCGA-HNSC | Larynx | Male |
| 43 | TCGA-CN-A497 | TCGA-HNSC | Larynx | Male |
| 44 | TCGA-CR-6474 | TCGA-HNSC | Larynx | Male |
| 45 | TCGA-CN-A49B | TCGA-HNSC | Larynx | Male |
| 46 | TCGA-TN-A7HJ | TCGA-HNSC | Larynx | Male |
| 47 | TCGA-CR-7402 | TCGA-HNSC | Larynx | Male |
| 48 | TCGA-CR-7371 | TCGA-HNSC | Larynx | Female |
| 49 | TCGA-CV-5430 | TCGA-HNSC | Larynx | Male |
| 50 | TCGA-CN-A63U | TCGA-HNSC | Larynx | Male |
| 51 | TCGA-CV-5432 | TCGA-HNSC | Larynx | Male |
| 52 | TCGA-QK-AA3J | TCGA-HNSC | Larynx | Male |
| 53 | TCGA-CV-7250 | TCGA-HNSC | Larynx | Male |
| 54 | TCGA-CV-5431 | TCGA-HNSC | Larynx | Male |
| 55 | TCGA-CV-7248 | TCGA-HNSC | Larynx | Female |
| 56 | TCGA-CV-7261 | TCGA-HNSC | Larynx | Male |
| 57 | TCGA-CN-A63W | TCGA-HNSC | Larynx | Female |
| 58 | TCGA-CN-4722 | TCGA-HNSC | Larynx | Female |
| 59 | TCGA-CV-7418 | TCGA-HNSC | Larynx | Male |
| 60 | TCGA-CN-A63T | TCGA-HNSC | Larynx | Male |
| 61 | TCGA-BA-6869 | TCGA-HNSC | Larynx | Male |
| 62 | TCGA-CV-A6K1 | TCGA-HNSC | Larynx | Male |
| 63 | TCGA-CR-7389 | TCGA-HNSC | Larynx | Male |
| 64 | TCGA-CV-7430 | TCGA-HNSC | Larynx | Male |
| 65 | TCGA-CR-7399 | TCGA-HNSC | Larynx | Female |
| 66 | TCGA-F7-7848 | TCGA-HNSC | Larynx | Male |
| 67 | TCGA-BA-A6DI | TCGA-HNSC | Larynx | Male |
| 68 | TCGA-KU-A66S | TCGA-HNSC | Larynx | Female |
| 69 | TCGA-D6-6517 | TCGA-HNSC | Larynx | Male |
| 70 | TCGA-D6-A6ES | TCGA-HNSC | Larynx | Male |
| 71 | TCGA-CV-7089 | TCGA-HNSC | Larynx | Male |
| 72 | TCGA-CV-A45Z | TCGA-HNSC | Larynx | Male |
| 73 | TCGA-F7-A623 | TCGA-HNSC | Larynx | Male |
| 74 | TCGA-CV-A461 | TCGA-HNSC | Larynx | Male |
| 75 | TCGA-CV-7424 | TCGA-HNSC | Larynx | Male |
| 76 | TCGA-F7-A50I | TCGA-HNSC | Larynx | Male |
| 77 | TCGA-UF-A7JK | TCGA-HNSC | Larynx | Male |
| 78 | TCGA-CV-6935 | TCGA-HNSC | Larynx | Male |
| 79 | TCGA-CN-4739 | TCGA-HNSC | Larynx | Male |
| 80 | TCGA-BA-A6DA | TCGA-HNSC | Larynx | Female |
| 81 | TCGA-QK-A8Z8 | TCGA-HNSC | Larynx | Female |
| 82 | TCGA-CV-7421 | TCGA-HNSC | Larynx | Male |
| 83 | TCGA-CV-A45Y | TCGA-HNSC | Larynx | Male |
| 84 | TCGA-UF-A7JJ | TCGA-HNSC | Larynx | Male |
| 85 | TCGA-CV-A460 | TCGA-HNSC | Larynx | Male |
| 86 | TCGA-CV-7242 | TCGA-HNSC | Larynx | Female |
| 87 | TCGA-CN-6010 | TCGA-HNSC | Larynx | Male |
| 88 | TCGA-F7-A622 | TCGA-HNSC | Larynx | Male |
| 89 | TCGA-D6-8568 | TCGA-HNSC | Larynx | Male |
| 90 | TCGA-UF-A7J9 | TCGA-HNSC | Larynx | Male |
| 91 | TCGA-D6-A6EQ | TCGA-HNSC | Larynx | Male |
| 92 | TCGA-CR-7364 | TCGA-HNSC | Larynx | Male |
| 93 | TCGA-CR-7388 | TCGA-HNSC | Larynx | Female |
| 94 | TCGA-BA-5555 | TCGA-HNSC | Larynx | Male |
| 95 | TCGA-CV-6962 | TCGA-HNSC | Larynx | Male |
| 96 | TCGA-QK-A8ZB | TCGA-HNSC | Larynx | Male |
| 97 | TCGA-CV-A45W | TCGA-HNSC | Larynx | Male |
| 98 | TCGA-T3-A92M | TCGA-HNSC | Larynx | Male |
| 99 | TCGA-F7-8298 | TCGA-HNSC | Larynx | Male |
| 100 | TCGA-CN-5360 | TCGA-HNSC | Larynx | Male |
| 101 | TCGA-CR-7374 | TCGA-HNSC | Larynx | Female |
| 102 | TCGA-D6-6824 | TCGA-HNSC | Larynx | Male |
| 103 | TCGA-BB-4217 | TCGA-HNSC | Larynx | Male |
| 104 | TCGA-CN-4738 | TCGA-HNSC | Larynx | Male |
| 105 | TCGA-CV-7247 | TCGA-HNSC | Larynx | Male |
| 106 | TCGA-CV-5435 | TCGA-HNSC | Larynx | Male |
| 107 | TCGA-BA-4078 | TCGA-HNSC | Larynx | Male |
| 108 | TCGA-CV-5978 | TCGA-HNSC | Larynx | Female |
| 109 | TCGA-CN-5361 | TCGA-HNSC | Larynx | Male |
| 110 | TCGA-CV-5434 | TCGA-HNSC | Larynx | Male |
| 111 | TCGA-UF-A7JH | TCGA-HNSC | Larynx | Male |
| 112 | TCGA-CV-7101 | TCGA-HNSC | Larynx | Male |
